# Validity testing of the Korean version of the Health Literacy Questionnaire (HLQ) and its application in people with chronic diseases

**DOI:** 10.1371/journal.pone.0308086

**Published:** 2024-08-01

**Authors:** Yon Hee Seo, Richard H Osborne, Yeunhee Kwak, Jung-Won Ahn

**Affiliations:** 1 Department of Nursing Science, Andong National University, Andong-si, Gyeongsangbuk-do, Korea; 2 Centre for Global Health and Equity, School of Health Sciences, Swinburne University of Technology, Hawthorn, Victoria, Australia; 3 Red Cross College of Nursing, Chung-Ang University, Seoul, Korea; 4 Department of Nursing, Gangneung-Wonju National University, Wonju-si, Gangwon-do, Korea; Tabriz University of Medical Sciences, ISLAMIC REPUBLIC OF IRAN

## Abstract

Health literacy plays a crucial role in promoting and maintaining the health of patients with chronic illnesses. Therefore, adequate assessments and the application of interventions based on people’s health literacy strengths, needs, and preferences are required to improve health outcomes. This study aimed to evaluate the psychometrical properties of the Health Literacy Questionnaire (HLQ) in Koreans with chronic diseases. Data were collected from 278 patients (57.04±15.22 years) diagnosed with chronic disease, including kidney disease, hypertension, and diabetes, who visited the outpatient clinic of a university hospital from June to December 2020. For validity assessment, construct, convergent, and discriminant validities were evaluated, along with the HLQ reliability using Cronbach’s α. One-way analysis of variance was used to evaluate mean differences in the HLQ scale scores based on patients’ characteristics. The confirmatory factor analysis (CFA) indicated that all items were loaded on their respective factors. The model fit of a full nine-factor CFA model showed satisfactory or better fit compared with nine one-factor CFA model; χ^2^_WLSMV_ (866) = 576.596 (*p* < .001), comparative normed fit index of 1.000 (reference: >0.950), Tucker–Lewis index of 0.981 (reference: >0.950), root mean square error of approximation of 0.066 (reference: <0.080), and standardized root mean square residual of 0.055 (reference: <0.080). All scales demonstrated good to excellent internal consistency (Cronbach’s α ≥.757). Sociodemographic characteristic variables with significant score differences in HLQ scores were reported across nine scales, with the level of education and income showing significant score differences in 8 and 6 scales, respectively. This study revealed that the Korean version of the HLQ has many strong measurement properties among patients with chronic diseases. The validation indicated the HLQ as a robust tool that is used cross-culturally and is recommended for use in the Korean population.

## Introduction

The prevalence of chronic diseases, such as hypertension, diabetes, and kidney disease, along with medical expenses, is increasing annually in Korea due to the aging population and a Westernized lifestyle [1]. A combination of genetic, physiological, environmental, and behavioral factors influences chronic diseases that are noncommunicable and persist over a long period. Lack of disease and risk factor management may result in complications and death [2].

The prevalence of hypertension, which is the most significant proportion of chronic diseases in Korea among individuals aged ≥20 years, increased by an average of 1.94 times in 2021 compared with that in 2007 [3]. The reported rate of antihypertensive medication use among adults with hypertension is approximately 58.6% [3], which is slightly lower than the rate in the United States at 59.6% [3, 4]. Interest in health literacy (HL) has been growing, considering its vital role in the health management of patients with complex and long-term chronic diseases [5, 6]. Individuals with chronic conditions benefit most from HL because it improves self-management and helps to sustain healthcare interventions. Higher HL enables people to make their own health decisions and respond to daily challenges. Previous studies have revealed that lower HL is associated with low confidence in lifestyle changes, less proactive health behaviors, inadequate healthcare utilization, denial of health problems, and poorer health outcomes compared with those having higher HL [7, 8]. Therefore, medical service providers should assess the HL levels of patients to effectively deliver health information and help patients with chronic diseases in successful health management [9].

Previous studies have frequently limited HL measurement to simple functional reading comprehension and numeracy skills [10–12]. The World Health Organization (WHO) indicated that individual’s HL encompasses knowledge, confidence, and comfort accumulated through daily activities and social interactions, enabling access, understanding, appraisal, memory, and use of health-related information for personal and collective well-being [13]. The WHO broadened the concept to include community HL and HL responsiveness to reflect interactions between people and their community and the surrounding systems and services [14].

An Australian global health team developed an assessment tool to measure the multiple dimensions of HL based on extensive consultation with community members and practitioners using a validity-driven approach to advance HL research [15]. The HL questionnaire (HLQ) comprises 44 items covering nine HL dimensions. This study considers cross-cultural health behaviors, management, and psychometric properties in general as well as comprehensive health management situations that are not limited to a particular race, language, or medical condition. The reliability and validity of the HLQ have been thoroughly verified, as it has been translated into >30 languages and used in >60 countries in Africa, Asia, America, Oceania, and Europe [15–18]. The psychometric properties of the HLQ in South Korea have been investigated among general healthy populations in the community setting [19] but not in individuals with chronic diseases, who are the primary targets of HL development programs.

This study aimed to comprehensively investigated the psychometric properties of the Korean version of the HLQ among key target populations, specifically people with chronic diseases, using health services in South Korea. These efforts ensure the availability of multidimensional HL tools that capture daily problems, improve HL development among individuals with chronic diseases in the community, and inform the responsiveness of their required health services.

## Materials and methods

### Study design

This cross-sectional study aimed to evaluate the validity and reliability of the HLQ [15] among Koreans diagnosed with chronic diseases and to describe the level of health literacy across sociodemographic groups of chronically ill patients.

### Participants

Participants were Koreans aged ≥18 years with chronic diseases who provided informed consent. Patients with mental illness or intellectual disability were excluded. Participants were recruited from outpatient clinics at a university hospital in Korea from June to December 2020. The target number of participants was considered suitable to develop a stable confirmatory factor analysis (CFA) [20]. The questionnaires were distributed to 300 patients, considering the dropout rate. Of the 285 returned questionnaires, 278 were analyzed, excluding data from seven participants with missing data.

### Measurements

#### Sociodemographic characteristics

Sociodemographic characteristics included participants’ sex, age, educational level, co-habitant, employment, and economic status. Furthermore, health-related characteristics, such as type of chronic condition, and experience of emergency admission within the last 12 months.

#### HLQ

The HLQ consisted of the following nine independent scales, each with 4–6 items, covering nine theoretically distinct areas of HL [15]:

1. Feeling understood and supported by healthcare providers (4 items)
2. Having sufficient information to manage my health (4 items)
3. Actively managing my health (5 items)
4. Seeking social support for health (5 items)
5. Appraising health information (5 items)
6. Actively engaging with healthcare providers (5 items)
7. Navigating the healthcare system (6 items)
8. Finding good health information (5 items)
9. Understanding health information well enough to know what to do (5 items)

The response options for scales 1–5 (Part 1) ranged from “1 (strongly disagree)” to “4 (strongly agree).” Scales 6–9 (part 2) contained five possible response options ranging from “1 (cannot do or always difficult)” to “5 (always easy).” Cronbach’s α value was.757–.910.

#### Perceived Kidney Disease Self-Management Scale (PKDSMS)

The hypothetical relationship was established to verify the convergent validity based on previous research results showing a weakly to moderately positive correlation relationship between self-management ability and health literacy in people with various chronic diseases [21–24]. Among the study participants, the self-management scores of patients with kidney disease (N = 230) were measured, and the correlation analysis was performed with the HLQ scores. This single-domain scale assessed self-management in CKD regardless of dialysis. The scale consists of eight items scored on a five-point Likert scale, including four inverse items. Higher scores indicate higher self-management levels. The Cronbach’s α of the scale was.76 at the time of its development [22] and.738 in this study.

#### Data collection

Permission for used the HLQ was obtained from the Swinburne University of Technology prior to the initiation of the study. A draft version of the Korean HLQ that was undergoing the validation process was provided by the original research team. Data were collected from July 25 to December 2, 2020. A preliminary survey was conducted with 7, 8, 4, and 2 Koreans in their 30s, 40s, 50s, 60s, and 70s, respectively. The aim was to monitor participants’ understanding of words, response times, and adjustments to enhance participants’ comprehension before proceeding to the main survey.

#### Ethical considerations

This study was conducted following the Institutional Review Board approval (1041078-202004-HR-114-01). All potential participants received an explanation about the purpose of the study, the coverage of participation, and the voluntariness of participation. All participants provided informed consent. Respondents anonymously provided data.

### Statistical analysis

Analyses were conducted using Mplus version 8.1 and IBM SPSS Statistical Package for the Social Sciences version 26.0 (IBM Corp., Armonk, NY, USA). The HLQ score was calculated following the Scoring Algorithm Instructions and syntax provided by the developer. The HLQ validation followed the guidelines of the consensus-based standards for the selection of health measurement instruments [25]. In Mplus, |skewness| <2 and |kurtosis| <7 indicates a multivariate normal distribution [26]. While the data in this study met the criteria for normality, we used weighted least-squares mean and variance-adjusted WLSMV estimation during the analysis. This decision was made to facilitate comparison with the findings of previous studies that validated the HLQ [16]. Nine one-factor CFA models and one nine-factor model were used to fit the data to confirm the scales, following the analysis methodology of a previous study [15]. Correlation analysis was performed to verify discriminant validity between latent variables by analyzing inter-factor relationships and the convergent validity within patients with kidney disease. χ^2^ was used to evaluate model fit for WLSMV estimation, degrees of freedom, comparative normed fit index (CFI), Tucker–Lewis index (TLI), root mean-squared error of approximation (RMSEA), and standardized root mean-square residual (SRMR). Values of <0.060 and <0.080 were interpreted as a reasonable fit for RMSEA and <0.080 for SRMR. A cut-off value of 0.900–0.950 was applied for both CFI and TLI [27].

The difficulty level was calculated as the fraction of “disagree/strongly disagree” responses against “agree/strongly agree” responses for scales 1–5. The difficulty level was calculated as the fraction of those who responded “cannot do or always difficult/usually difficult/sometimes difficult” compared with “usually easy/always easy.” for scales 6–9. The difference in the health literacy for different groups of demographic and health status was examined by t-test or two-way analysis of variance. Effect sizes and their 95% confidence intervals for standardized differences in means between sociodemographic groups were calculated as the small effect size (.20–.50) and medium effect size (.50–.80), following the analysis methodology of a previous study [16].

## Results

### Participant characteristics

The participants had an average age of 57.04 ± 15.22 (range 19–89) years, 152 (54.7%) were male, and 189 (68.0%) were high school graduates or less. Additionally, 53 (19.1%) were living alone and 106 (38.1%) were employed during the study. Moreover, 88 (31.7%) had visited the emergency room (ER) in the preceding 12 months. Among those with chronic diseases, renal disease (37.8%), hypertension (29.2%), and diabetes (20.4%) were the most prevalent (Table 1).

**Table 1:** Characteristics of the participants (N = 278).

Characteristics	Categories	n (%)
Sex	Male	152 (54.7)
	Female	126 (45.3)
Age (years, range: 19–89)	<45	56 (20.2)
	45–64	133 (47.8)
	≥65 years	89 (32.0)
Education level	Middle school or lower	75 (27.0)
	High school	114 (41.0)
	College or above	89 (32.0)
Living alone	Yes	53 (19.1)
	No	225 (80.9)
Currently working	Yes	106 (38.1)
	No	172 (61.9)
Income	<$900	57 (20.5)
	$900–1,799	56 (20.1)
	$1,800–2,699	68 (24.5)
	$2,700–3,599	42 (15.1)
	$3,600 or more	55 (19.8)
Emergency room admission within 12 months	Yes	88 (31.7)
	No	190 (68.3)
Type of chronic conditions*	Kidney disease	230 (37.8)
(n = 609)	Hypertension	178 (29.2)
	Diabetes	124 (20.4)
	Neurovascular disease	31 (5.1)
	Cardiac disease	24 (3.9)
	Other	22 (3.6)

*Individuals may report more than one disease

### Psychometric properties of the HLQ

Table 2 shows the psychometric properties of the HLQ items and scales. The level of difficulty level of the items ranged from a low value of 7.2 (scale 4, item 15) to 72.3 (scale 8, item 18). The scales with the highest average difficulty were “8: Finding good health information” (66.5), “7: Navigating the healthcare system” (63.7), and “6: Actively engaging with healthcare providers” (55.8), and the scales with the lowest reported difficulties were “4” Seeking social support for health” (16.5), “1: Feeling understood and supported by healthcare providers” (25.6), and “5: Appraising health information” (35.9).

**Table 2:** Difficulty level of the items and psychometric properties of the HLQ^a^ using the one-factor and nine-factor CFA^b^ models (N = 278).

Scales/Item		Mean ± SD^c^	Difficulty (95% CI^d^)	Average item difficulty (%)	One-factor CFA	
					Standardized estimates (95% CI)	R^2^
**Part 1: Scales 1–5: how strongly you disagree or agree with the following statements**						
**1. Feeling understood and supported by healthcare providers**						
2	I have at least one healthcare… knows…	2.78 ± 0.71	28.1 (22.9–33.7)	25.6	.71 (.63–78)	.50
8	I have at least one healthcare… discuss…	2.89 ± 0.63	18.3 (14.0–23.4)		.78 (.72–.84)	.60
17	I have the healthcare providers… help me…	2.63 ± 0.72	37.8 (32.0–43.8)		.77 (.70–.83)	.59
22	I can rely on at least one…	2.91 ± 0.63	18.0 (13.7–23.0)		.79 (.73–.85)	.63
**Mean ± SD**		2.80 ± 0.55				
Model fit (one-factor)–χ^2^_WLSMV_^e^ (2) = 5.208, *p* < .001, CFI^f^ = 0.993, TLI^g^ = 0.978, RMSEA^h^ = 0.076, SRMR^i^ = 0.015						
Cronbach’s α = .842, Composite reliability = .844						
**2. Having sufficient information to manage my health**						
1	I feel that I have good information…	2.65 ± 0.63	35.3 (29.6–41.2)	52.0	.58 (.49–.68)	.34
10	I have enough information to help me…	2.49 ± 0.63	49.6 (43.6–55.7)		.67 (.58–.76)	.45
14	I have all the information I need…	2.31 ± 0.69	63.3 (57.3–69.0)		.69 (.60–.77)	.47
23	I have all the information I need…	2.39 ± 0.68	59.7 (53.7–65.5)		.75 (.67–.85)	.56
**Mean ± SD**		2.46 ± 0.50				
Model fit (one-factor)–χ^2^_WLSMV_ (2) = 0.919, *p* < .001, CFI = 1.000, TLI = 1.000, RMSEA = 0.000, SRMR = 0.009;						
Cronbach’s α = .757, Composite reliability = .763						
**3. Actively managing my health**						
6	I spend quite a lot of time actively…	2.65 ± 0.70	38.5 (32.7–44.5)	39.7	.71 (.64–.78)	.51
9	I make plans for what I need to do…	2.53 ± 0.71	42.8 (36.9–48.9)		.73 (.66–.80)	.53
13	Despite…, I make time to be healthy	2.70 ± 0.72	35.6 (30.0–41.6)		.74 (.67–.81)	.55
18	I set my goals for health and fitness	2.54 ± 0.67	44.6 (38.7–50.7)		.74 (.67–.81)	.55
21	There are things that I do regularly…	2.68 ± 0.69	37.1 (34.4–43.0)		.72 (.64–.79)	.51
**Mean ± SD**		2.62 ± 0.55				
Model fit (one-factor)–χ^2^_WLSMV_ (5) = 25.283, *p* < .001, CFI = 0.962, TLI = 0.924, RMSEA = 0.077, SRMR = 0.030						
Cronbach’s α = .844, Composite reliability = .844						
**4. Seeking social support for health**						
3	I can get access to several people…	2.91 ± 0.67	20.1 (15.6–25.3)	16.5	.64 (.55–.73)	.41
5	When I feel ill, the people around me…	2.99 ± 0.59	12.9 (9.2–17.5)		.68 (.59–.76)	.46
11	If I need help, I have plenty of people…	2.81 ± 0.66	29.1 (23.9–34.9)		.72 (.64–.80)	.52
15	I have at least one person who can come	3.14 ± 0.56	7.2 (4.4–10.9)		.55 (.45–.66)	.31
19	I have strong support from family or friends.	3.03 ± 0.55	13.3 (9.5–17.9)		.71 (.63–.80)	.51
**Mean ± SD**		2.97±0.45				
Model fit (one-factor)–χ^2^_WLSMV_ (5) = 42.451, *p* < .001, CFI = .906, TLI = 0.881, RMSEA = 0.071, SRMR = 0.053						
Cronbach’s α = .789, Composite reliability = .709						
**5. Appraising health information**						
4	I compare health information from different…	2.56 ± 0.72	39.6 (33.8–45.6)	35.9	.66 (.58–.74)	.44
7	When I see new health information…	2.63 ± 0.71	37.4 (31.7–43.4)		.74 (.66–.81)	.54
12	I always compare health information…	2.71 ± 0.67	30.2 (24.9–36.0)		.77 (.70–.84)	.59
16	I know how to find out if the health information I receive is …	2.62 ± 0.69	39.9 (34.1–45.9)		.63 (.54–.72)	.39
20	I ask healthcare providers… the quality of…	2.69 ± 0.65	32.4 (26.9–38.2)		.60 (.51–.69)	.36
**Mean ± SD**		2.64±0.52				
Model fit (one-factor)–χ^2^_WLSMV_ (5) = 13.730, *p* = .017, CFI = 0.979, TLI = 0.958, RMSEA = 0.079, SRMR = 0.028						
Cronbach’s α = .804, Composite reliability = .808						
**Part 2: Scales 6–9: How easy or difficult the following tasks are for you to do now**						
**6. Actively engaging with healthcare providers**						
2	Make sure that healthcare providers understand…	3.25 ± 0.86	58.3 (52.2–64.1)	55.8	.76 (.70–.82)	.58
4	Feel able to discuss health concerns with…	3.26 ± 0.95	54.3 (48.3–60.3)		.81 (.76–.86)	.66
7	Have good discussions about your health…	3.44 ± 0.91	48.2 (42.2–54.2)		.72 (.65–.78)	.51
15	Discuss… until you understand	3.11 ± 1.00	61.9 (55.9–67.6)		.82 (.77–.87)	.67
20	Ask healthcare providers questions to get the…	3.28 ± 0.90	56.5(50.4–62.4)		.74 (.68–.80)	.55
**Mean ± SD**		3.27 ± 0.76				
Model fit (one-factor)–χ^2^_WLSMV_ (5) = 21.018, *p* < .001, CFI = 0.977, TLI = 0.953, RMSEA = 0.063, SRMR = 0.025						
Cronbach’s α = .879, Composite reliability = .882						
**7. Navigating the healthcare system**						
1	Find the right healthcare	3.10 ± 0.93	66.5 (60.7–72.1)	63.7	.65 (.58–.72)	.42
8	Get to see the healthcare providers I need to…	3.26 ± 1.05	52.5 (46.5–58.5)		.81 (.76–.86)	.66
11	Decide which healthcare provider…	2.93 ± 0.96	71.6 (65.9–76.8)		.80 (.75–.85)	.64
13	Make sure you find the right place…	3.27 ± 0.98	55.4 (49.3–61.3)		.85 (.80–.89)	.71
16	Find out what healthcare services you are…	3.04 ± 1.00	66.2 (60.3–71.7)		.84 (.79–.88)	.70
19	Work out what is the best care for you	2.99 ± 0.94	70.1 (64.4–75.5)		.71 (.65–.78)	.51
**Mean ± SD**		3.10±0.80				
Model fit (one-factor)–χ^2^_WLSMV_ (9) = 24.735, *p* = .003, CFI = 0.983, TLI = 0.972, RMSEA = 0.079, SRMR = 0.024						
Cronbach’s α = .900, Composite reliability = .907						
**Scale 8. Finding good health information**						
3	Find information about health problems	3.06 ± 0.95	64.7 (58.8–70.4)	66.5	.83 (.79–.88)	.69
6	Find health information from several different…	3.03 ± 1.01	64.4 (58.4–70.0)		.83 (.79–.88)	.69
10	Get information about health so you are…	3.07 ± 1.00	63.3 (57.3–69.0)		.83 (.79–.88)	.70
14	Get health information in words you understand	3.03 ± 0.99	68.0 (62.2–73.4)		.78 (.72–.83)	.60
18	Get health information by yourself	2.83 ± 1.03	72.3 (66.6–77.5)		.82 (.78–.87)	.67
**Mean ± SD**		3.00 ± 0.86				
Model fit (one-factor)–χ^2^_WLSMV_ (5) = 12.424, *p* = .029, CFI = 0.992, TLI = 0.983, RMSEA = 0.073, SRMR = 0.016						
Cronbach’s α = .910, Composite reliability = .908						
**Scale 9. Understanding health information well enough to know what to do**						
5	Confidently fill medical forms…	3.23 ± 1.06	54.0 (47.9–59.9)	54.9	.72 (.66–.80)	.54
9	Accurately follow the instructions…	3.56 ± 0.88	42.8 (36.9–48.9)		.62 (.49–.67)	.33
12	Read and understand written health information	3.22 ± 0.99	56.8 (50.8–62.7)		.79 (.76–.87)	.66
17	Read and understand all the information on…	2.90 ± 1.05	71.2 (65.5–76.5)		.71 (.67–.81)	.54
21	Understand what healthcare providers are…	3.39 ± 0.86	49.6 (43.6–55.7)		.72 (.63–.77)	.49
**Mean ± SD**		3.26 ± 0.75				
Model fit (one-factor)–χ^2^_WLSMV_ (5) = 16.981, *p* = .005, CFI = 0.976, TLI = 0.953, RMSEA = 0.047, SRMR = 0.028						
Cronbach’s α = .834, Composite reliability = .842						
**Model fit (nine-factor)**–**χ**^**2**^_**WLSMV**_ **(866) = 576.596, *p* < .001, CFI = 1.000, TLI = 0.981, RMSEA = 0.066, SRMR = 0.055**						

^a^HLQ: health literacy questionnaire

^b^CFA: confirmatory factor analysis

^c^SD: standard deviation

^d^CI: confidence interval

^e^WLSMV: weighted least-squares mean and variance adjusted

^f^CFI: comparative fit index

^g^TLI: Tucker–Lewis index

^h^RMSEA: root mean-square error of approximation

^i^SRMR: standardized root mean-square residual

One-factor CFA demonstrated that the model fit of the nine scales met the recommended criteria. The CFA revealed that all one-factor models generally demonstrated good model fit indices, except for scale 4, where the TLI was.881. The composite reliability was good to excellent for all scales (≥.709), and Cronbach’s α was.757–.910.

The full nine-factor CFA showed good-to-excellent factor loadings, better than the one-factor model and all items loading on their respective factors ≥.50 were satisfied. The full nine-factor CFA model fitted to the 44 items, with no cross-loadings or correlated residuals. The fit was relatively satisfactory: χ^2^_WLSMV_ (866) = 576.596, *p* < .001, CFI = 1.000, TLI = 0.981, RMSEA = 0.066, SRMR = 0.055 (Table 2).

### Discrimination and convergent validity testing of the HLQ

The inter-factor correlation coefficients between the nine HLQ factors ranged from.120 to.935. The correlation index between scales 1 and 8 was.120 and that between scales 1 and 9 was.172. The correlation indices between scales 7, 8, and 9 were.918 to.935 (Table 3).

**Table 3:** Factor correlations of the health literacy questionnaire (N = 278).

**Scale**	**Part 1: Scales 1–5**					**Part 2: Scales 6–9**		
	**1** ^a^	**2** ^b^	**3** ^c^	**4** ^d^	**5** ^e^	**6** ^f^	**7** ^g^	**8** ^h^
	***r* (*p*)**								
2	.556**							
3	.514**	.798**						
4	.651**	.527**	.521**					
5	.440**	.904**	.815**	.383**				
6	.435**	.529**	.405**	.477**	.364**			
7	.246**	.598**	.333**	.364**	.483**	.858**		
8	.120*	.609**	.326**	.273**	.545**	.746**	.935**	
9	.172**	.519**	.367**	.323**	.459**	.883**	.927**	.918**

** < .01,

* < .05

^a^1: feeling understood and supported by healthcare providers

^b^2: having sufficient information to manage health

^c^3: actively managing my health

^d^4: seeking social support for health

^e^5: appraising health information

^f^6: actively engaging with healthcare providers

^g^7: navigating the healthcare system

^h^8: finding good health information

^i^9: understanding health information well enough to know what to do

To test the hypothetical relationship, the correlation between the PKDSMS scores and the HLQ scores in patients with kidney disease was analyzed for convergent validity testing. Scales 1–5 (*r* = .502, *p* < .001) and 6–9 (*r* = .411, *p* < .001) of HLQ with PKDSMS showed statistically significant correlation coefficients.

### Differences in the HLQ scores between participant characteristics

The scores on scale 1 “Healthcare providers’ support” were higher in the groups aged ≥65 years, with lower than high school education, and are unemployed. The scores for scale 2 “Having sufficient information” were low in the groups with high school education or lower and <$900 income. The scale 3 “Actively managing health” scores were significantly higher in the groups aged ≥65 years and college education or higher. The scale 4 “Seeking social support” scores were higher in the group aged ≥65 years, not living alone, and ≥$2,700 income than <$900. The groups with college education or higher and ≥$900 income have higher scores on scale 5 “Appraising health information.” No significant differences were found in the general characteristics for scale 6 “Actively engaging with healthcare providers,” The scores on scale 7 “Navigating the healthcare system” were significantly higher in the groups with a college education, employed, ≥$900 income, and no ER admissions recorded within the preceding 12 months. The scores on scale 8 “Finding good health information” were significantly increased for males aged <65 years, with college education or higher, employed, and with higher income levels. The scores on scale 9 “Understanding health information” were significantly higher in the groups with high school education, employed, ≥$2,700 income than <$900 income, and no ER admission recorded within the preceding 12 months (Table 4).

**Table 4:** Relationships between patient characteristics of patients and disease and health literacy questionnaire scores (N = 278).

			1. Feeling understood and supported by healthcare providers	2. Having sufficient information to manage health	3. Actively managing my health	4. Seeking social support for health	5. Appraising health information	6. Actively engaging with healthcare providers	7. Navigating the healthcare system	8. Find good health information	9. Understanding health information
Characteristics	Categories	n (%)				Mean ± SD					
			2.80 ± 0.55	2.46 ± 0.50	2.62 ± 0.55	2.97 ± 0.45	2.64 ± 0.52	3.27 ± 0.76	3.10 ± 0.80	3.00 ± 0.86	3.26 ± 0.75
Sex	Male	152(54.7)	2.76 ± 0.60	2.50 ± 0.52	2.64 ± 0.55	2.98 ± 0.48	2.68 ± 0.55	3.33 ± 0.73	3.17 ± 0.77	3.11 ± 0.85	3.33 ± 0.76
	Female	126(45.3)	2.85 ± 0.49	2.41 ± 0.48	2.60 ± 0.55	2.96 ± 0.41	2.60 ± 0.48	3.20 ± 0.80	3.01 ± 0.83	2.87 ± 0.84	3.17 ± 0.74
	Difference		-0.90	0.08	0.05	0.02	0.08	0.13	0.16	0.24	0.17
	Effect size (95% CI)		-.16(-.40|.07)	.12(-.07|.40)	.12(-.15|.32)	.12(-.18|.29)	.12(-.08|.40)	.12(-.07|.41)	.12(-.03|.44)	.12(.05|.52)*	.12(-.01|.46)
Age (year)	<65 years	189(68.0)	2.70 ± 0.53	2.44 ± 0.46	2.57 ± 0.53	2.92 ± 0.44	2.65 ± 0.46	3.25 ± 0.75	3.15 ± 0.72	3.10 ± 0.77	3.30 ± 0.70
	≥65 years	89(32.0)	3.02 ± 0.54	2.50 ± 0.58	2.72 ± 0.58	3.10 ± 0.44	2.63 ± 0.63	3.32 ± 0.78	2.98 ± 0.95	2.80 ± 0.98	3.17 ± 0.85
	Difference		-0.32	-0.06	-0.15	-0.18	0.01	-0.07	0.17	0.30	0.14
	Effect size (95% CI)		-.57(-.82|-.33) *	-.12(-.38|.13)	-.27(-.52|.02)*	-.40(-.65|-.15)*	.03(-.23|.28)	-.09(-.35|.16)	.21(-.07|.49)	.35(.08|.63)*	.18(-.09|.45)
Education level	< High school^a^	75(27.0)	2.94 ± 0.53	2.37 ± 0.51	2.59 ± 0.54	3.05 ± 0.38	2.52 ± 0.56	3.17 ± 0.81	2.78 ± 0.88	2.49 ± 0.84	2.94 ± 0.78
	High school^b^	114(41.0)	2.71 ± 0.51	2.36 ± 0.50	2.54 ± 0.54	2.90 ± 0.42	2.58 ± 0.51	3.26 ± 0.71	3.09 ± 0.77	2.99 ± 0.80	3.28 ± 0.73
	≥ College^c^		2.81 ± 0.61	2.65 ± 0.46	2.75 ± 0.56	3.00 ± 0.52	2.82 ± 0.46	3.36 ± 0.78	3.38 ± 0.66	3.45 ± 0.67	3.49 ± 0.68
	Difference a versus b		0.23	0.00	0.05	0.15	-0.06	-0.09	-0.31	-0.51	-0.34
	Effect size (95% CI)		.41(.15|.67) *	.01(-.29|.30)	.08(-.20|.37)	.34(.07|.60)*	-.11(-.41|.19)	-.11(-.40|.18)	.39(-.68|-.09) *	.14(-.88|-.32) *	.15(-.74|-.16) *
	Difference a versus c		0.13	-0.29	-0.16	0.05	-0.30	-0.19	-0.60	-0.97	-0.54
	Effect size (95% CI)		.23(-.09|.55)	-.57(-.87|-.27) *	-.29(-.60|.03)	.10(-.22|.42)*	-.57(-.87|-.27) *	-.25(-.57|.08)	-.75(-1.05|-.44)*	-1.13(-1.41|-.86)*	.11(-.77|-.32) *
	Difference b versus c		-0.10	-0.29	-0.20	-0.11	-0.24	-0.10	-0.29	-0.46	-0.20
	Effect size (95% CI)		-.19(-.47|.09)	-.58(-.84|-.31) *	-.37(-.64|-.09) *	-.24(-.53|.05)*	-.46(-.72|-.20) *	-.13(-.40|.14)	-.36(-.61|-.11) *	.54(-.78|-.29) *	.27(-.53|-.01)
Living alone	Yes	53(19.1)	2.72 ± 0.58	2.47 ± 0.48	2.63 ± 0.57	2.82 ± 0.45	2.69 ± 0.45	3.29 ± 0.78	3.14 ± 0.84	3.15 ± 0.82	3.35 ± 0.77
	No	225(80.9)	2.82 ± 0.55	2.45 ± 0.51	2.62 ± 0.55	3.01 ± 0.44	2.63 ± 0.53	3.26 ± 0.76	3.09 ± 0.79	2.97 ± 0.86	3.24 ± 0.75
	Difference		-0.10	0.02	0.01	-0.19	0.06	0.03	0.05	0.18	0.11
	Effect size (95% CI)		-.18(-.48|.12)	.03(-.27|.34)	.01(-.29|.31)	-.43(-.73|-.14)*	.11(-.19|.41)	.04(-.26|.34)	.06(-.24|.36)	.21(-.09|.51)	.15(-.15|.45)
Currently working	Yes	106(38.1)	2.66 ± 0.58	2.45 ± 0.46	2.60 ± 0.53	2.95 ± 0.46	2.69 ± 0.41	3.31 ± 0.73	3.28 ± 0.65	3.26 ± 0.70	3.42 ± 0.68
	No	172(61.9)	2.89 ± 0.52	2.47 ± 0.53	2.63 ± 0.56	2.98 ± 0.44	2.61 ± 0.57	3.25 ± 0.78	2.99 ± 0.86	2.84 ± 0.90	3.16 ± 0.78
	Difference		-0.23	-0.02	-0.03	-0.03	0.08	0.06	0.29	0.42	0.26
	Effect size (95% CI)		-.42(-.66|-.18)*	-.04(-.28|.21)	-.06(-.30|.18)	-.07(-.31|.18)	.15(-.08|.37)	.08(-.16|.32)	.37(.14|.59)*	.49(.27|.71)*	.34(.10|.58)*
Income	<$900^a^	57(20.5)	2.77 ± 0.59	2.21 ± 0.55	2.48 ± 0.67	2.81 ± 0.52	2.34 ± 0.61	3.09 ± 0.88	2.72 ± 0.86	2.60 ± 0.91	3.01 ± 0.83
	$900–2699^b^	124(44.6)	2.83 ± 0.48	2.52 ± 0.47	2.67 ± 0.49	2.99 ± 0.37	2.74 ± 0.45	3.30 ± 0.70	3.17 ± 0.81	2.97 ± 0.81	3.26 ± 0.72
	≥$2700^c^	97(34.9)	2.78 ± 0.60	2.53 ± 0.47	2.63 ± 0.52	3.04 ± 0.47	2.68 ± 0.47	3.33 ± 0.75	3.22 ± 0.68	3.28 ± 0.77	3.40 ± 0.72
	Difference a versus b		-0.07	-0.33	-0.20	-0.18	-0.40	-0.22	-0.46	-0.38	-0.25
	Effect size (95% CI)		-.12(-.42|.18)	-.65(-.96|-.34)*	-.37(-.69|-.07)	-.41(-.75|-.07)*	-.77(-1.08|-.46)*	-.29(-.60|.03)	-.58(-.90|-.25)*	-.45(-.76|-.13)*	-.34(-.65|-.02)*
	Difference a versus c		-0.01	-0.30	-0.15	-0.22	-0.34	-0.24	-0.50	-0.68	-0.39
	Effect size (95% CI)		-.01(-.37|.35)	-.60(-.92|-.27)*	-.28(-.63|.07)	-.49(-.85|-.13)*	-.65(-.99|-.32)*	-.31(-.66|.03)	-.62(-.93|-.31)*	-.80(-1.12|-.48)*	-.52(-.86|-.19)*
	Difference b versus c		0.06	0.03	0.05	-0.04	-0.06	-0.02	-0.38	-0.30	-0.14
	Effect size (95% CI)		0.11(-.15|.37)	0.05(-.20|.30)	0.09(-.16|.34)	-0.08(-.33|.17)	0.12(-.12|.36)	-0.03(-.28|.23)	-0.05(-.30|.21)	-0.35(-.60|-.10)*	-0.19(-.44|.07)
ER admission within 12 months	Yes	88(31.7)	2.83 ± 0.60	2.45 ± 0.56	2.67 ± 0.61	2.96 ± 0.52	2.66 ± 0.57	3.16 ± 0.77	2.94 ± 0.83	2.89 ± 0.87	3.09 ± 0.75
	No	190(68.3)	2.79 ± 0.53	2.46 ± 0.48	2.60 ± 0.52	2.98 ± 0.41	2.63 ± 0.49	3.32 ± 0.75	3.17 ± 0.78	3.06 ± 0.85	3.34 ± 0.74
	Difference		0.03	-0.01	0.07	-0.02	0.03	-0.16	-0.23	-0.17	-0.25
	Effect size (95% CI)		.06(-.19|.31)	-.01(-.26|.25)	.13(-1.28|.38)	-.05(-.30|.21)	.04(-.21|.30)	-.21(-.46|.04)	-.29(-.54|-.04)*	-.20(-.45|.05)	-.33(-.58|-.08)*

*p < .05

## Discussion

This study examined the HL levels of patients with chronic diseases across nine HLQ factors based on general characteristics. The results of the correlation analysis among nine factors in this study showed that the factor was between 1/8 and 1/9 and the correlation coefficient between factors was < .2. Although there was consistent report on low correlation (*r* = .18) between 1/9 [18], the result of correlation of ≥.3 between all other factors was reported in many previous studies [17, 28], which confirmed discriminant validity, whereas scales 7–9 had correlation coefficients of ≥0.80. Therefore, future studies must repeat correlational analysis to test the discriminant validity of the HLQ within the Korean population. Low to medium correlations with self-management of patients with renal disease were reported among various chronic diseases [21–24], and the discriminant validity (*r* ≥.40) was confirmed in this study.

The reliability of the HLQ was evaluated using internal consistency, with internal consistency value of.757–.910. This result was similar to the results of the following studies: developmental research that reported Cronbach’s α of.87–.90 [15], a Danish study reporting Cronbach’s α of.77–.87 [16], a Slovakian study with Cronbach’s α of.73–.84 [29], and a Chinese verification study with Cronbach’s α of.74–.85 [18]. Thus, the internal consistency of the HLQ was confirmed. The difficulty level analysis revealed the lowest and highest difficulty levels on scales 4 and 2 in Part 1 and scales 9 and 8 in Part 2, respectively.

The participants demonstrated high HL levels necessary for social support and functional literacy, but they had difficulty gaining access to adequate information and information searching. This may be attributed to patients with chronic diseases preferring specific and high-quality information over health information required by the general population. A study in which participants with recent hospitalization experiences had low HL levels for the respective aspects of HL supported this inference [30]. The order of the scores for each item was similar to those of previous studies, but the overall difficulty level was high [16]. In particular, the difficulty level of Part 2 was higher than that indicated in some European and Australian studies [16]. However, no significant differences were found in the results of this study when compared with those of existing Asian studies, including Chinese and Vietnamese [18, 31]. The low score for mutual HL, such as using health information and navigating healthcare systems, may be due to differences in the perception of the role of healthcare providers. Asian cultures recognize healthcare providers as therapists, whereas the role of health managers is mainly emphasized in Western cultures. Therefore, future studies should investigate the effects of cultural differences in HL and each aspect of HL. There is a growing need for Korean medical professionals to expand their role to serve as healthcare managers for individual patients, rather than adhering solely to their traditional role as medical personnel.

The HL analysis by characteristics revealed that the same group faced challenges in information literacy although the HL levels in terms of healthcare providers and social support were high in those aged ≥65 years. The score for social support was high, particularly in those aged ≥65 years, possibly because Confucian culture influences South Korean values, which emphasizes a family-centered philosophy and children’s responsibility to support their parents. Meanwhile, the score on scale 4 was significantly lower among single-person household participants, consistent with previous studies [16]. Those with a university/college education or higher demonstrated high HL scores in six areas; this finding is consistent with those of previous studies [16]. Additionally, HL levels were high among participants with higher incomes, which may be because many Korean public health medical systems are private. A previous study revealed that the average number of diseases increased as respondent ages increased and their socioeconomic status, such as level of education and income, decreased. Additionally, they were more likely to report poor self-rated health and require higher medical services. However, participants with high socioeconomic status were less likely to access medical services [32], indicating that socioeconomic status is one of the main causes of health inequality [33]. Education level and socioeconomic status were associated with issues such as difficulty in self-management of chronic diseases, interaction with healthcare professionals, and use of health education resources [34, 35]. In this study, participants with ER admissions within the preceding 12 months scored poorly on medical system navigation and functional literacy. This finding is consistent with those of previous studies in that the low HL group was highly associated with a high admission rate, use of emergency medical services, neglect of preventive medical services, noncompliance with medication, negative self-rated health, chronic disease co-occurrence, and unhealthy behaviors [36, 37]. Therefore, policy support is required for continuous health education and the allocation of medical resources to resolve health inequality issues.

Health information is based on expert-level knowledge and scientific evidence and verified by experts in related fields, but its value and social effect are determined by recipients’ levels of understanding of information [38]. Recently, methods of obtaining health information—a core component of health information literacy—have become increasingly diverse. Specifically, obtaining health information online has become common with the widespread use of smartphones [39, 40]. The rate of Internet use even among individuals aged ≥60 years increased from 69.5% in 2015 to 88.8% in 2018, and from 17.9% in 2015 to 38.6% in 2018 among those in their 70s [41, 42]. Therefore, development various interventional approaches related to online health information would be effective. However, individuals who search for health information online are easily exposed to a vast volume of unverified health information, and the misuse of unfiltered health information negatively affects the user’s health [39, 41]. Individuals find it difficult to determine the health information that applies to them because of rapidly advancing medical technology and an overwhelming influx of health-related information. Therefore, medical professionals and institutions are required to participate and intervene in the provision, understanding, use, and linkage of quality information and medical resources, focusing on recipients.

The study has some limitations. The results are difficult to generalize because participant data were collected at more than one hospital, and the characteristics of participants’ chronic diseases could have influenced the results. Also, the period and severity of illness in chronically ill patients were not included in detail and could not be distinguished. The attitudes of healthcare providers at the participating hospitals could have affected the results. Additionally, data can be skewed when patients with chronic diseases have developed a good rapport with healthcare providers because of the characteristics of patients with chronic diseases.

The study’s findings highlight the importance of establishing an information support system through local governments to facilitate the access of information about the selection of medical practitioners and healthcare facilities by the people with chronic diseases. At the community level, it appears critical to target priority groups, such as those <65 years, living alone, with low income and education levels, and those who are employed, to comprehensively address their specific needs. As significant, researchers developed and verified the HLQ to investigate the HL levels of patients with chronic diseases across nine HLQ factors that represent the concept of HL as a multidimensional construct, as defined by the WHO. The content can be revised appropriately to each culture context, if necessary, and retested for validity by verifying scales 1/8 and 1/9, which had low correlation following the correlational analysis between factors. Further studies are warranted to determine the suitability of the HLQ in various types of medical conditions and age groups. Based on the results of the HL evaluation, a systematic strategy to promote individual HL through intervention plans can be established for each area of HL.

## Conclusion

The examination of HLQ reliability and validity in patients with chronic diseases revealed high levels of construct validity, and overall reliability. The HLQ may provide multidimensional HL evaluation results for various population groups, including patients with chronic diseases. The results of this study will provide a reference for research on HL in patients with chronic diseases as well as policy development and understanding of their health.

## Supporting information

S1 Data (XLSX)
